# Structural Characterization of Heme Environmental Mutants of CgHmuT that Shuttles Heme Molecules to Heme Transporters

**DOI:** 10.3390/ijms17060829

**Published:** 2016-05-27

**Authors:** Norifumi Muraki, Chihiro Kitatsuji, Mariko Ogura, Takeshi Uchida, Koichiro Ishimori, Shigetoshi Aono

**Affiliations:** 1 Institute for Molecular Science, National Institutes of Natural Sciences, 5-1 Higashiyama, Myodaiji, Okazaki 444-8787, Japan; nmuraki@ims.ac.jp (N.M.); bb20114202@cc.nagasaki-u.ac.jp (C.K.); 2 Okazaki Institute for Integrative Bioscience, National Institutes of Natural Sciences, 5-1 Higashiyama, Myodaiji, Okazaki 444-8787, Japan; 3 Graduate School of Chemical Sciences and Engineering, Hokkaido University, Sapporo, Hokkaido 060-8628, Japan; m6_ed4_science@ec.hokudai.ac.jp (M.O.); uchida@sci.hokudai.ac.jp (T.U.); koichiro@sci.hokudai.ac.jp (K.I.); 4 Department of Chemistry, Faculty of Science, Hokkaido University, Sapporo, Hokkaido 060-0810, Japan; 5 Department of Structural Molecular Science, SOKENDAI, The Graduate University for Advanced Studies, 5-1 Higashiyama, Myodaiji, Okazaki 444-8787, Japan

**Keywords:** heme uptake, heme transport, substrate binding protein in ATP-binding cassette (ABC) transporter, X-ray crystallography

## Abstract

Corynebacteria contain a heme uptake system encoded in *hmuTUV* genes, in which HmuT protein acts as a heme binding protein to transport heme to the cognate transporter HmuUV. The crystal structure of HmuT from *Corynebacterium glutamicum* (CgHmuT) reveals that heme is accommodated in the central cleft with His141 and Tyr240 as the axial ligands and that Tyr240 forms a hydrogen bond with Arg242. In this work, the crystal structures of H141A, Y240A, and R242A mutants were determined to understand the role of these residues for the heme binding of CgHmuT. Overall and heme environmental structures of these mutants were similar to those of the wild type, suggesting that there is little conformational change in the heme-binding cleft during heme transport reaction with binding and the dissociation of heme. A loss of one axial ligand or the hydrogen bonding interaction with Tyr240 resulted in an increase in the redox potential of the heme for CgHmuT to be reduced by dithionite, though the wild type was not reduced under physiological conditions. These results suggest that the heme environmental structure stabilizes the ferric heme binding in CgHmuT, which will be responsible for efficient heme uptake under aerobic conditions where Corynebacteria grow.

## 1. Introduction

Heme can be used as an iron source in some bacteria, for which a heme uptake system is required. Gram-negative and Gram-positive bacteria utilize different heme uptake machineries [1,2,3,4,5]. In Gram-negative bacteria, heme uptake machineries consist of three components: a receptor protein at the outer membrane, a periplasmic heme-binding protein, and an ABC-type heme transporter at the inner membrane [4,5]. The receptor proteins bind heme-containing proteins or heme-bound hemophore and transport heme into the periplasm in a TonB-dependent manner via an energy-driven process [4,5]. Once the heme is transported into the periplasm, the heme is bound by a periplasmic heme-binding protein to be shuttled to an ATP-binding cassette (ABC)-type heme transporter in the inner membrane. An ABC-type heme transporter consisting of a permease and an ATPase is responsible for heme transport from periplasm to cytoplasm.

In Gram-positive bacteria, heme captured by cell-wall-anchored heme receptor proteins is transferred to a membrane-bound ABC-type heme transporter to be transported into cytoplasm (Figue S1 in Supplementary Materials). Among Gram-positive bacteria, the heme uptake machinery of *Staphylococcus aureus*, the Iron-regulated surface determinant system (Isd), has been studied in detail [6,7,8,9]. Four cell-wall-anchored proteins (IsdABCH) and a membrane-bound ABC-type heme transporter complex (IsdDEF) play a crucial role for the heme uptake by the Isd system [6,7,8,9]. IsdA, IsdB, IsdC, and IsdH act as heme (IsdA and IsdC), hemoglobin (IsdB), and hemoglobin-haptoglobin (IsdH) receptors [10]. They contain at least one near iron transporter (NEAT) domains capable of binding one heme molecule [8,11,12,13]. IsdB and IsdH contain two and three NEAT domains, respectively, while IsdA and IsdC contain one NEAT domain each [8]. Heme is transferred unidirectionally among these Isd proteins [14,15,16]. Heme captured in the NEAT domain 2 of IsdB is transferred to the NEAT domains of either IsdA or IsdC. Heme transfer from IsdA to IsdC is also unidirectional [8,14,15,16]. IsdC acts as a node for heme transfer from the heme receptor proteins to IsdE, which is a heme-binding protein for an ABC-type heme transporter IsdDF [8,14,15,16].

**Figure 1:**
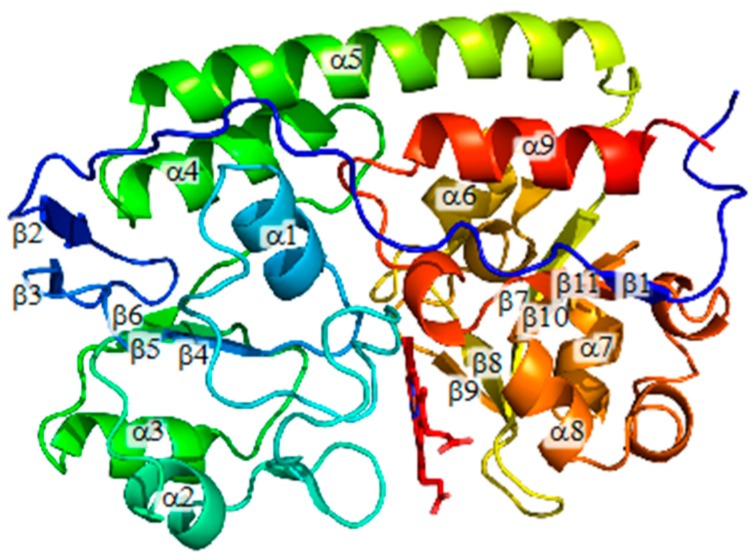
Overall structure of HmuT from *Corynebacterium glutamicum* (CgHmuT).

While *Bacillus anthracis* utilizes a similar Isd system for heme uptake [17,18], another Gram-positive bacteria like *Corynebacterium diphtheriae* and *Corynebacterium glutamicum* contain a different heme uptake system consisting of HtaA, HtaB, and HmuTUV [19,20,21], as shown in Figure 1. HtaA and HtaB are membrane-bound heme receptor proteins containing two and one conserved region (CR) responsible for heme binding, respectively [21]. HmuTUV forms an ABC-type heme transporter system, in which HmuT, HmuU, and HmuV are the heme binding lipoprotein, permease, and ATPase, respectively. HtaA has been reported to interact with hemoglobin (Hb) to bind heme extracted from Hb [21]. Heme bound in HtaA is transferred to HtaB and finally transferred to the HmuT that shuttles heme to the ABC-type heme transporter HmuUV [20,21]. Group A Streptococcus like *Streptococcus pyogenes* utilizes similar heme acquisition machinery consisting of Shr, Shp, and the ABC-type heme transporter HtsABC [22]. Shr has two NEAT domains and binds hemoglobin and the hemoglobin-haptoglobin complex [23,24]. Heme bound in Shr is transferred to Shp. Shp transports heme to HtsA acting as a substrate-binding protein in the ABC-transporter to transfer heme into the heme permease [25].

Though Gram-negative and Gram-positive bacteria utilize different heme uptake systems, they share similar ABC-type heme transporters to transport heme into cytoplasm. In fact, the crystal structure of HmuT from *Corynebacterium glutamicum* (CgHmuT) reveals that it shares a similar global structure with HmuT from a Gram-negative bacterium *Yersinia pestis* (YpHmuT), which is a heme-binding component of the ABC-type heme transporter system [26]. Heme molecules captured by HmuT are transferred to a heme permease protein and finally transported into cytoplasm. CgHmuT consists of two lobes connected with a α-helix, both of which consist of a five-stranded β-sheet and four α-helices [26]. There is a cleft to accommodate a heme molecule between two lobes. His141 and Tyr240 are coordinated to the heme as the axial ligands and Arg242 forms a hydrogen bond with Tyr240 in CgHmuT [26].

As HmuT plays an important role for heme uptake, the elucidation of the structure and function relationships of HmuT is required to understand the detailed molecular mechanisms of the heme uptake process. The regulation of heme binding/dissociation properties of CgHmuT is crucial for an efficient heme uptake process. Though the heme environmental structure including axial ligation and hydrogen bonding interaction between Tyr240 and Arg242 is thought to regulate heme binding/dissociation properties of CgHmuT, their biological functions remain to be elucidated. In this work, X-ray crystallographic and spectroscopic analyses have been done for CgHmuT mutants in which His141, Tyr240, or Arg242 is replaced by Ala to understand the regulatory mechanisms of heme binding/dissociation in CgHmuT.

## 2. Results

All three mutants of CgHmuT (H141A-, Y240A-, and R242A-CgHmuT) were predominantly expressed as a holo form in *Escherichia coli (E. coli)* cells, indicating that the heme binding ability of these mutants are retained. The purified heme-bound proteins were crystallized, and the crystal structures of H141A-, Y240A-, and R242A-CgHmuT were determined in the holo-form at the resolution of 1.30, 1.65, and 1.30 Å, respectively. A summary of the data collection and refinement statistics are shown in Table 1.

The overall structure of wild-type CgHmuT is shown in Figure 1, which is similar to that of H141A-, Y240A-, and R242A-CgHmuT (Figue S2 in Supplementary Materials). The root-mean square (r.m.s.) deviation between wild-type and H141A-, Y240A-, or R242A-HmuT for all C_α_ atoms were 0.15, 0.20, and 0.18 Å, respectively. These CgHmuT mutants also consist of two globular domains connected to a long α-helix, as is the case of wild-type CgHmuT. The two globular domains share a similar topology consisting of a five-stranded β-sheet and four α helices, between which there is a cleft to accommodate a heme molecule. Though heme is accommodated with two different orientations in the heme-binding cleft in wild-type CgHmuT, it was not the case in all mutants. While two different orientations of heme were observed in Y240A-CgHmuT, heme was accommodated with a single orientation in H140A- and R242A-CgHmuT.

**Figure 2:**
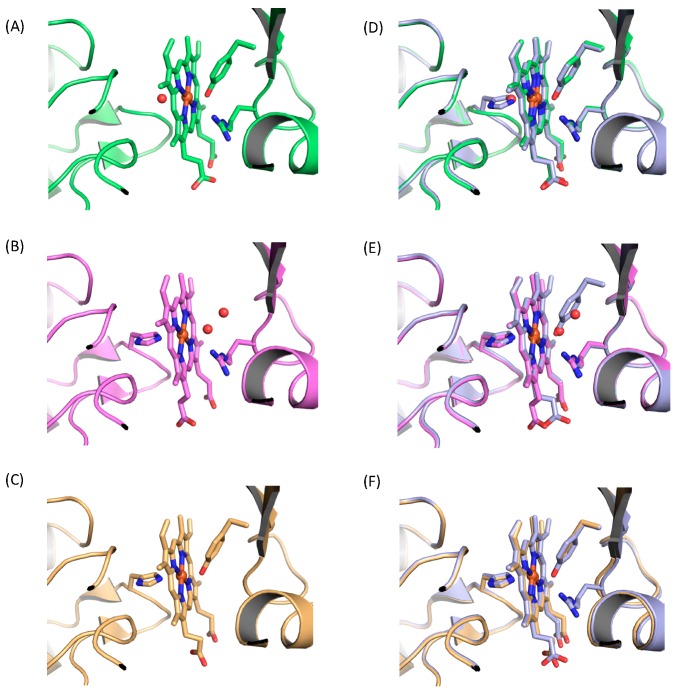
Heme environmental structure of (**A**) H141A mutant (green), (**B**) Y240A mutant (magenta), and (**C**) R242A mutant (gold). Structural comparison between wild-type (WT) (gray) and (**D**) H141A mutant, (**E**) Y240A mutant, and (**F**) R242A mutant. A red ball represents a water molecule.

The comparison of heme environmental structures between wild-type and H141A mutants is shown in Figure 2A,D. In H141A-CgHmuT, the axial ligation of Tyr240 and the hydrogen bond between Tyr240 and Arg242 were retained. The distance between the oxygen atom of the OH group in Tyr240 and iron was 2.0 Å, which was identical to that for wild-type CgHmuT. The side chains of Tyr240 and Arg242 were entirely superimposable between wild-type and H141A mutants. On the other hand, the conformation of the loop composed from Gly139 to Ala141 was slightly perturbed by the mutation of His141 to Ala, while other parts were almost superimposable between them. A water molecule was observed at the same position as that of the nitrogen atom of His141 in wild-type CgHmuT. The distance between the water and the iron was 2.6 Å.

**Figure 3:**
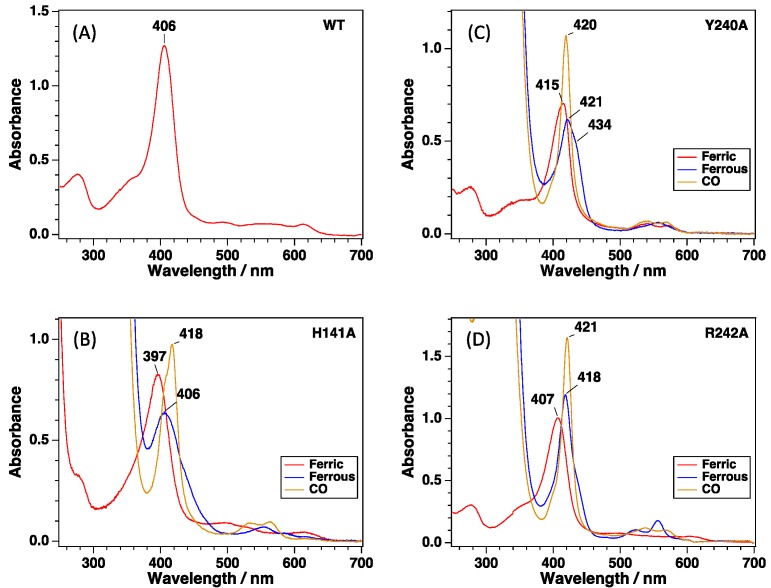
Electronic absorption spectra of (**A**) wild-type (WT), (**B**) H141A-, (**C**) Y240A-, and (**D**) R242A-CgHmuT. The only ferric spectrum is shown for wild-type CgHmuT because it is not reduced by dithionite.

Ferric wild-type CgHmuT showed the Soret and a charge transfer (CT) band at 406 and 614 nm, respectively, with a broad absorption in the Q-band region (Figure 3A). The electronic absorption spectrum of the ferric CgHmuT did not change upon adding sodium dithionite, indicating that the reduction of heme in CgHmuT does not proceed, probably due to a low redox potential of the heme. The ferric H141A-CgHmuT showed the Soret peak and a single broad Q-band at 397 and 495 nm, respectively Figure 3B). The Soret peak was blue-shifted by 9 nm compared with wild-type ferric CgHmuT. The charge transfer band at 614 nm was also observed, which is typical for hemeproteins that tyrosine is coordinated to heme as an axial ligand. Though wild-type ferric CgHmuT is not reduced by dithionite, probably due to a low redox potential of the heme, H141A-CgHmuT was reduced by dithionite. The Soret peak was shifted to 406 nm upon reduction, and the Q-band peaks were observed at 555 and 585 nm in the ferrous H141A-CgHmuT. Reaction of ferrous H141A-CgHmuT with CO resulted in the formation of the CO-bound form, which showed the Soret, α, and β peaks at 418, 564 and 530 nm, respectively (Figure 3B).

**Figure 4:**
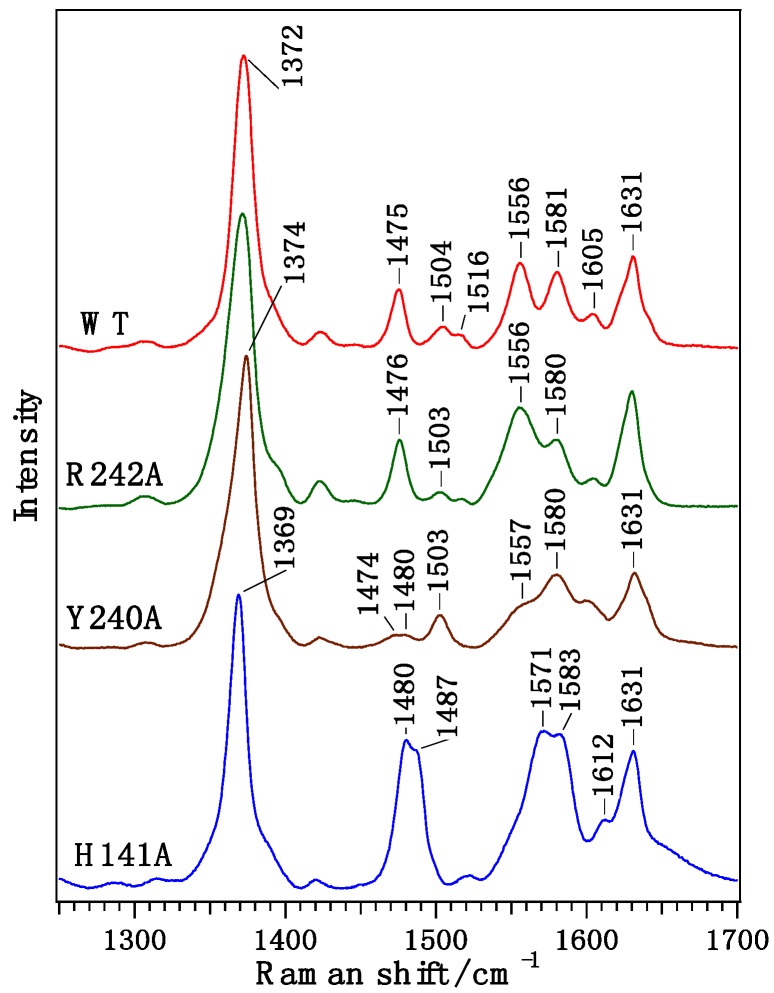
Resonance Raman spectra of WT, H141A, Y240A, and R242A CgHmuT in the high frequency region.

Ferric H141A-CgHmuT showed the ν_2_, ν_3_, and ν_4_ bands at 1571 and 1583, 1487 and 1480, and 1369 cm^−1^, respectively, in the high frequency resonance Raman spectra (Figure 4). The ν_2_ and ν_3_ bands at 1583 and 1487 cm^−1^, respectively, are due to a five-coordinate high spin heme, while those at 1571 and 1480 cm^−1^ are due to a six-coordinate high spin heme. These results indicate that both the 5cHS and 6cHS species are present in ferric H141A-CgHmuT.

The crystal structure of H141A-CgHmuT reveals that a water molecule is present near the heme iron, suggesting that the 6cHS and 5cHS species observed in solution are attributed to the heme coordinated with Tyr/H_2_O and Tyr, respectively. The distance 2.6 Å between the water and iron in H141A-CgHmuT crystal seems to be long for a coordinate bond, which suggests that the observed structure corresponds to the 5cHS species. The equilibrium will be present in the H141A-CgHmuT solution for the coordination/dissociation of water to/from the heme iron.

The heme environmental structure of Y240A-CgHmuT is shown in Figure 2B. Except for Tyr240, there was little difference in its structure including the conformation of His141 and Arg242 compared with wild-type (Figure 2E). His141 was coordinated to the heme iron with 2.2 Å of bond distance. Two water molecules were present in the heme pocket, where Tyr240 is located in wild-type. The oxygen atom of proximal water molecule in Y240A-CgHmuT was superimposable to the oxygen atom of Tyr240 in wild-type when comparing two structures. The distances were 2.2 and 2.7 Å between the oxygen atom of proximal water molecule and iron, and between that and the nitrogen atom of the NH group in Arg242, respectively. These results indicate that the proximal water molecule is coordinated to the heme iron as an axial ligand with the formation of a hydrogen bond with Arg242, as is the case for Tyr240 in wild-type.

Ferric Y240A-CgHmuT showed the Soret, α, and β peaks at 415, 575 and 540 nm, respectively, which was a typical electronic absorption spectrum of six-coordinate, low-spin (6cLC) heme with a histidine as one axial ligand (Figure 3C). This mutant was also reduced by dithionite. Ferrous Y240A-CgHmuT showed the Soret and single Q-band peaks at 421 and 558 nm, respectively. Ferrous Y240A-CgHmuT reacted with CO to result in the formation of the CO-bound form, showing the Soret, α, and β peaks at 420, 570 and 540 nm, respectively (Figure 3C).

As shown in Figure 4, ferric Y240A-CgHmuT showed the ν_2_ and ν_3_ bands at 1580 and 1503 cm^−1^, respectively, which are due to a 6cLS heme. The ν_2_ (1557 cm^−1^) and ν_3_ (1474 and 1480 cm^−1^) bands due to a 6cHS heme were also observed as a minor species. Ferric M79A-HtsA that contains a 6cHS heme with His and a water as its axial ligands shows the ν_2_ and ν_3_ bands at 1562 and 1478 cm^−1^, respectively [27]. Though the coordination of His/water is identical between Y240A-CgHmuT and M79A-HtsA, the spin states of them are different from each other. The 6cLS state is predominant in Y240A-CgHmuT, which will be caused by the hydrogen bonding interaction between the heme-bound water and Arg242. This hydrogen bonding interaction may increase the OH^−^ character of the heme-bound water.

Figure 2C,F shows the structural comparison between wild-type and R242A mutants. There was no perturbation in the heme environmental structure in R242A-CgHmuT except for the absence of the Arg242 compared with wild-type. The bond distances between the heme iron and the nitrogen atom and the oxygen atom of His141 and Tyr240 were 2.1 and 2.0 Å, respectively. Though the axial ligation with His141 and Tyr240 was retained in R242A-CgHmuT as wild-type, the redox property was different from each other. R242A-CgHmuT was reduced by dithionite, unlike wild-type. The Soret peak of R242A-CgHmuT was shifted from 407 to 418 nm upon reduction. Ferrous R242A-CgHmuT showed the α and β peaks at 556 and 523 nm, respectively (Figure 3D).

As shown in Figure 4, ferric R242A-CgHmuT showed the ν_2_ bands at 1556 and 1581 cm^−1^ and the ν_3_ bands at 1476 and 1503 cm^−1^, which were identical to those of the wild type. The set of the ν_2_ and ν_3_ bands at 1556 and 1476 cm^−1^ are due to a high-spin heme, while those at 1581 and 1503 cm^−1^ are due to a low-spin heme. These results indicate that there is equilibrium between the high-spin and low-spin species in the R242A-CgHmuT solution, as is the case of the wild type.

## 3. Discussion

As CgHmuT is a substrate (heme) binding protein that shuttles heme to the cognate ABC-type heme transporter CgHmuUV in Corynebacteria [19], the regulation of heme binding and release is very important for its physiological function. The crystal structure of wild-type CgHmuT reveals that the specific recognition and binding of heme is achieved by axial ligation of the heme with His141 and Tyr240, though hydrophobic environment of the heme-binding cleft seems to play a role for stabilizing heme accommodation [26]. In this work, we have characterized H141A-, Y240A-, and R242A-CgHmuT by X-ray crystallography and resonance Raman spectroscopy to elucidate functional roles of His141, Tyr240, and Arg242 for heme recognition.

Protein structure database searching by the Dali server [28] has revealed that CgHmuT shows a structural homology to periplasmic substrate binding proteins that shuttle transporting substrates to the cognate ABC-type transporters. CgHmuT showed a homology to YpHmuT [29], PhuT [30], and ShuT [24] with a *Z*-score of >24 and the RMSD of <2.7 Å for all C_α_ atoms between them and CgHmuT, indicating that these structures are very similar. The structural fold of these proteins is typical for the Type III periplasmic binding protein (PBP) [31,32], which is characterized by little conformational change upon binding the cognate substrate to apo protein. While the opening-closing of two lobes in response to the substrate-dissociation/binding is observed in Type I and Type II PBPs such as the maltose-binding protein MalE [33], the motion associated with heme binding is smaller in YpHmuT [29]. In ShuT, the most obvious difference between holo- and apo-forms is in the orientation of the Tyr67 heme ligand [30]. Though there is little difference in the protein conformation, there are two orientations of Tyr67:Tyr67 either points “in” toward the heme pocket, where it is in position to coordinate the heme iron, or it flips out toward the surface [30]. Though the structure of apo CgHmuT is not determined yet, the crystal structures of H141A- and Y240A-CgHmuT suggest that CgHmuT shares the characteristic structural property of Type III PBPs in that there is little conformational change upon binding the cognate substrate to apo protein.

An opening of the substrate binding cleft is observed for BtuF, which is the Type III PBP transporting vitamin B12 only when BtuF binds to the cognate ABC-type transporter BtuCD [34]. When the BtuCDF complex is formed, the conserved glutamates of BtuF interact with patches of conserved arginine residues of the permease BtuC [35]. The interactions between these glutamates and arginine residues are crucial for BtuCDF complex formation, which may cause a conformational change of BtuF. These glutamates are conserved in the Type III PBPs such as YpHmuT and FhuD2 in similar locations to those in BtuF. CgHmuT also contains glutamates—Glu146 and Glu284—at the corresponding positions, suggesting a similar regulatory mechanism in which the complex formation between CgHmuT and CgHmuUV causes an opening of the heme-binding cleft to promote heme release from CgHmuT.

Though the structural perturbation is not observed by the mutation of His141, Tyr240, or Arg242 in CgHmuT, the redox property of the heme in CgHmuT is affected by these mutations. While the heme bound to wild-type CgHmuT is not reduced by dithionite under normal conditions, the heme in H141A-, Y240A-, and R242A-CgHmuT are reduced by dithionite. These results show that the ferric heme is stabilized in CgHmuT by not only the coordination of His and Tyr as the axial ligands but also the hydrogen bonding interaction between the axial ligand Tyr and Arg. Removing hydrogen-bonding interactions between Ser92 and the proximal His ligand in myoglobin results in an increase in the redox potential of heme from 95 to 123 mV [36]. The electron donating ability of the axial ligand reduces by increasing the positive character of the proximal His caused by a loss of the hydrogen bond, which leads to an increase in the redox potential of heme [36]. The change in the redox property of R242A-CgHmuTA will be caused by a similar effect upon a loss of hydrogen bond between Tyr240 and Arg242. As CgHmuT works at the cell surface in aerobic conditions, ferric heme will be a predominant species for CgHmuT. Thus, the preference of CgHmuT to ferric heme will be responsible for efficient heme uptake.

## 4. Materials and Methods

Expression vectors for CgHmuT mutants were prepared by the protocol of QuickChange Site-Directed Mutagenesis Kit (Stratagene, La Jolla, CA, USA) using the expression vector of wild-type CgHmuT as a template. Expression and purification of CgHmuT mutants were carried out as those of wild-type CgHmuT [26]. All of the mutants, which consisted of Ala24-Glu359, were expressed with a C-terminal His_6_ tag as is the case of wild-type CgHmuT.

Crystallization of CgHmuT mutants was done via a hanging drop vapor diffusion method at 20 °C. The crystal of H141A mutant was obtained using 2.25 M ammonium sulfate, 0.2 M potassium thiocyanate, 0.2 M ammonium tartrate, and 0.025% (*w*/*v*) octyl β-d-glucopyranoside as a precipitant. The crystal of Y240A mutant was obtained using 2.1 M ammonium sulfate, 0.2 M potassium thiocyanate, 20% (*v*/*v*) glycerol, and 0.5% (*w*/*v*) octyl β-d-glucopyranoside as a precipitant. The crystal of H141A mutant was obtained using 1.9 M ammonium sulfate, 0.16 M potassium thiocyanate, and 0.19 M ammonium tartrate as a precipitant. For data collection under a cryogenic condition, the protein crystals were briefly soaked in a reservoir solution containing 15% (*v*/*v*) glycerol as a cryoprotectant. Diffraction data were obtained at 100 K on beamline BL44XU at SPring-8 (Hyogo, Japan). All diffraction data were processed with the programs iMosflm [37] and XDS [38], and scaled with the program Scala in the CCP4 suite and the program XSCALE [38,39]. The structure of CgHmuT mutants were determined by rigid-body refinement of the crystal structure of wild-type CgHmuT (PDB ID: 5AZ3), lacking heme as a starting model with the program Refmac5 in the CCP4 suite [40]. The manual model building was carried out with the program Coot [41]. The model was refined with the program Refmac5 [40] and Phenix.refine [42]. The statistics of data collection and refinement are summarized in Table 1. The atomic coordinates and structural factors have been deposited in the Protein Data Bank Japan as the PDB code of 5B4Z, 5B50, and 5B51 for H141A, Y240A, and R242A mutants, respectively.

Resonance Raman spectra were measured as previously reported [43,44,45]. Briefly, resonance Raman spectra were obtained by an excitation with a 413.1-nm line of a krypton ion laser (BeamLok 2060; Spectra Physics, Santa Clara, CA, USA) with a liquid nitrogen-cooled CCD detector (Spec-10:400B/LN; Roper Scientific, Trenton, NJ, USA) attached to a monochromator (SPEX500M; Horiba-Jobin Yvon, Kyoto, Japan). The Raman shifts were calibrated with indene, carbon tetrachloride, acetone, and an aqueous solution of ferrocyanide. The accuracy of the peak positions of the Raman bands was ±1 cm^−1^.

## Figures and Tables

**Table 1:** A summary of the data collection and refinement statistics.

CgHmuT Mutants	H141A	Y240A	R242A
**Data Collection**			
Space group	*P*4_1_2_1_2	*P*4_1_2_1_2	*P*4_1_2_1_2
Cell dimensions			
*a*, *b* (Å)	73.38	73.01	73.06
*c* (Å)	147.42	145.96	147.07
α, β, γ (°)	90.00	90.00	90.00
Wavelength (Å)	0.9000	0.9000	0.9000
Resolution (Å)	48.94–1.30 (1.34–1.30) ^1^	35.41–1.65 (1.74–1.65) ^1^	42.27–1.30 (1.34–1.30) ^1^
Observed Reflection	935723	1416608	889463
Unique Reflection	97636	48329	96893
*R*merge ^2^	0.058 (0.551) ^1^	0.093 (0.508) ^1^	0.069 (0.487) ^1^
*R*meas	0.062 (0.584) ^1^	0.097 (0.527) ^1^	0.073 (0.517) ^1^
*I*/σ*I*	19.98 (3.73) ^1^	16.60 (5.2) ^1^	17.52 (4.58) ^1^
Completeness (%)	98.8 (96.6) ^1^	100 (100) ^1^	98.3 (98.3) ^1^
**Refinement**			
Resolution (Å)	48.94–1.30	35.41–1.65	42.27–1.30
*R*work/*R*free (%)	15.6/17.3	17.6/20.7	16.3/18.1
No. of atoms			
Protein	2314	2403	2413
Heme	43	86	43
Water	230	141	225
Average B factors	14.8	23.3	16.5
**Root-mean-square deviations**			
Bond lengths (Å)	0.005	0.015	0.007
Bond angles (°)	1.153	1.391	1.352

^1^ Values in parentheses are for the highest resolution shells; ^2^ *R*merge(I) = Σ|*I*(*k*) − <*I*>|/Σ*I*(*k*), where *I*(*k*) is the value of the *k-*th measurement of the intensity of a reflection, <*I*> is the mean value of the intensity of that reflection and the summation is over all measurement.

## Supplementary Materials

Supplementary materials can be found at http://www.mdpi.com/1422-0067/17/6/829/s1.

## Abbreviations

The following abbreviations are used in this manuscript:

ABC	ATP-Binding Cassette
ATPase	adenosine triphosphatase
CO	carbon monoxide
CT	charge transfer
5cHS	five-coordinate high-spin
Hb	hemoglobin
Isd	iron-regulated surface determinant
NEAT	near iron transporter
r.m.s.	root-mean-square
6cHS	six-coordinate high-spin
6cLS	six-coordinate low-spin
