# Supplementary material for: Structural Characterization of Heme Environmental Mutants of CgHmuT that Shuttles Heme Molecules to Heme Transporters
Source: Int J Mol Sci. 2016 May 27;17(6):829. doi: 10.3390/ijms17060829 (PMC4926363; doi:10.3390/ijms17060829)
Supplement: Supplementary file 1 [file ijms-17-00829-s001.pdf]

Int. J. Mol. Sci. **2016**, *17*, 829; doi:10.3390/ijms17060829 www.mdpi.com/journal/ijms

Supplementary Materials: Structural Characterization of Heme Environmental Mutants of CgHmuT that Shuttles Heme Molecules to Heme Transporters

Norifumi Muraki, Chihiro Kitatsuji, Mariko Ogura, Takeshi Uchida, Koichiro Ishimori and Shigetoshi Aono

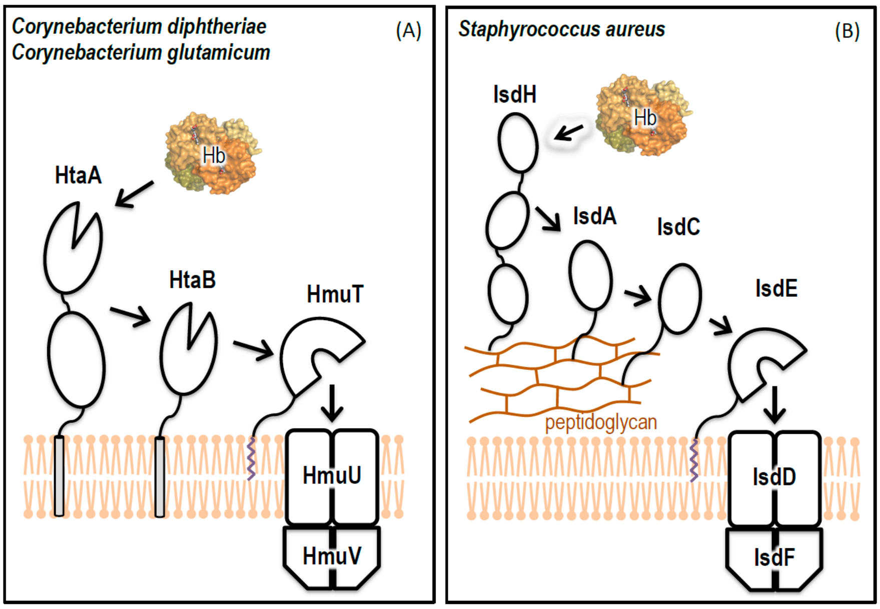

Figure S1. Model of heme acquisition system in (**A**) *Corynebacterium diphtheriae* and *Corynebacterium glutamicum* and (**B**) *Staphyrococcus aureus*.

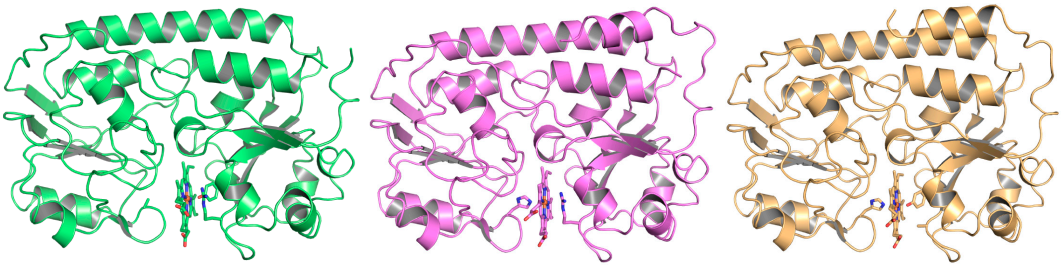

Figure S2. Overall structure of (**Left**) H141A, (**Middle**) Y240A, and (**Right**) R242A mutants.
